# Transcriptome analysis of basic fibroblast growth factor treated stem cells isolated from human exfoliated deciduous teeth

**DOI:** 10.1016/j.heliyon.2020.e04246

**Published:** 2020-06-25

**Authors:** Nunthawan Nowwarote, Jeeranan Manokawinchoke, Kiattipan Kanjana, Benjamin P.J. Fournier, Waleerat Sukarawan, Thanaphum Osathanon

**Affiliations:** a Center of Excellence for Regenerative Dentistry, Faculty of Dentistry, Chulalongkorn University, Bangkok 10330 Thailand; b Department of Anatomy, Faculty of Dentistry, Chulalongkorn University, Bangkok 10330 Thailand; c Genomics and Precision Dentistry Research Unit, Faculty of Dentistry, Chulalongkorn University, Bangkok 10330 Thailand; d Centre de Recherche des Cordeliers, Université de Paris, INSERM, Sorbonne Université, Molecular Oral Physiopathology, Paris, France; e Faculty of Dentistry Garanciere, Universite de Paris, France; f Department of Pediatric Dentistry, Faculty of Dentistry, Chulalongkorn University, Bangkok 10330 Thailand

**Keywords:** Cell biology, Bioinformatics, Transcriptomics, Biomedical engineering, Molecular biology, Regenerative medicine, Basic fibroblast growth factor, Transcriptome, RNA sequencing, Stem cells isolated from human exfoliated deciduous teeth

## Abstract

**Background:**

Basic fibroblast growth factor (bFGF) regulates cell proliferation, migration, and differentiation in various cell types. The aim of the present study was to determine the bFGF target genes in stem cells isolated from human exfoliated deciduous teeth (SHEDs).

**Methods:**

Cells were isolated from pulp tissue obtained from exfoliated deciduous teeth. Mesenchymal stem cell surface markers and the differentiation potential toward adipogenic and neurogenic lineages were characterized. The bFGF-treated SHED transcriptome was examined using a high throughput RNA sequencing technique. The mRNA and protein expression of selected genes were evaluated using real-time polymerase chain reaction and immunofluorescence staining, respectively. Cell cycle analysis was performed by flow cytometry. The colony forming unit number was also examined.

**Results:**

The isolated cells expressed CD44, CD90, CD105, but not CD45. The upregulation of adipogenic and neurogenic marker genes was observed after culturing cells in the appropriate induction medium. Transcriptome analysis of the bFGF treated cells revealed that the upregulated genes were in the cell cycle related pathways, while the downregulated genes were in the extracellular matrix related pathways. Correspondingly, bFGF induced *MKI6*7 mRNA expression and Ki67 protein expression. Furthermore, bFGF treatment significantly decreased the G0/G1, but increased the G2/M, population in SHEDs. Colony formation was markedly increased in the bFGF treated group and was attenuated by pretreating the cells with FGFR or PI3K inhibitors.

**Conclusion:**

bFGF controls cell cycle progression in SHEDs. Thus, it can be used to amplify cell number to obtain the amount of cells required for regenerative treatments.

## 1 Introduction

Stem cells from exfoliated deciduous teeth (SHEDs) are considered to be an alternative source of mesenchymal stem cells because they can be non-invasively isolated from the dental pulp tissue of exfoliated primary teeth. SHEDs have self-renewal and multi-differentiation ability [1]. After maintaining SHEDs in the appropriate induction medium, the cells differentiated into cell lineages derived from ectoderm, mesoderm, and endoderm [1,2]. In addition to their multi-differentiation potency, SHEDs exhibit immunomodulatory properties. SHEDs regulate monocyte-derived dendritic cell differentiation and maturation as well as inhibit CD4^+^ and CD8^+^ T cell proliferation [3]. SHEDs exhibit higher proliferation compared with other sources of mesenchymal stem cells, [4,5]. Transplanting SHEDs in murine calvarial defects resulted in a similar amount of bone regeneration compared with human dental pulp stem cells (hDPSCs) and human bone marrow mesenchymal stem cells (hBMSCs) [6]. However, the highest osteoid area was observed in the defects treated with SHEDs [6]. SHEDs combined with collagen scaffolds promoted dental pulp tissue formation and newly deposited tubular dentin in subcutaneously implanted root canals [7]. Based on these findings, SHEDs have been proposed as an alternative mesenchymal stem cell source for regenerative therapy, especially in the dental field.

Basic fibroblast growth factor (bFGF) is a member of the fibroblast growth factor family [8]. It regulates various cell responses, including cell proliferation, cell migration, and cell differentiation [8,9,10]. bFGF is commonly added to growth medium to maintain human embryonic or induced stem cells in an undifferentiated pluripotent state [11]. bFGF improved skin wound healing efficacy in diabetic mice based on an increased epithelization rate and cell proliferation [12]. Continuous treatment with bFGF suppressed osteogenic differentiation as determined by decreased alkaline phosphatase enzymatic activity and mineralization *in vitro* [13]. However, a randomized controlled trial indicated that a high concentration of recombinant bFGF combined with β-tricalcium phosphate (β-TCP) enhanced clinical attachment and bone fill in infrabony vertical periodontal defects compared with β-TCP alone [14]. It has been postulated that when used clinically, bFGF promotes cell migration and cell proliferation as well as enhances angiogenesis in the defect area, resulting in improved overall periodontal regeneration [14].

SHEDs express a significantly higher *bFGF* mRNA level compared with hDPSCs and hBMSCs [15,16]. bFGF upregulates the expression of several pluripotent markers, including *OCT4A*, *REX1*, and *NANOG* in SHEDs [10]. Mechanistically, it has been shown that bFGF regulates *REX1* expression via interleukin 6 (IL-6) [9]. Furthermore, bFGF enhances cell proliferation, colony forming unit number, and SHED migration *in vitro* [10,17]. bFGF inhibits odonto/osteogenic differentiation and mineralization in SHEDs by activating the ERK1/2 pathway and regulating phosphate/pyrophosphate regulatory genes [18,19]. In neurogenic induction, bFGF is a crucial growth factor supplement in neurobasal medium to induce neuronal differentiation in SHEDs [20].

These findings led to the hypothesis that SHEDs utilize different pathways in response to bFGF to control specific functions, such as proliferation, and multipotency, However, despite the extensive investigation into the effects of bFGF, the pathways and target genes regulated by bFGF in oral stem cells remain to be elucidated. Therefore, the aim of the present study was to investigate the entire bFGF-treated SHED transcriptome to identify regulatory pathways and their functions.

## 2 Materials and methods

### 2.1 Cell isolation and culture

The study protocol was approved by the Human Research Ethics Committee, Faculty of Dentistry, Chulalongkorn University (Approval number 079/2018). Human deciduous teeth treatment planned for extraction (e.g. exfoliation or prolong retention) were obtained for cell isolation. Teeth with pathological conditions were excluded from the study. The teeth were obtained from the Department of Pediatric Dentistry, Faculty of Dentistry, Chulalongkorn University. Informed consent was obtained. A standard explant protocol was used for cell isolation [21,22]. Briefly, the pulp tissue was gently removed from pulp chamber using barbed broach and cut into small pieces. The cut tissue was then placed on 35 mm tissue culture dish with culture medium, allowing cells to migrate out from tissues. After 7 days, cells and remaining tissues were trypsinized. The remaining tissue was discarded and the cells were reseeded in 60 mm tissue culture dish. The cells were maintained in Dulbecco's modified Eagle medium (DMEM Cat. No. 11960-044, Gibco™, ThermoFisher, NY, USA) supplemented with 2mM L-glutamine (GlutaMAX™-1 Cat. No. 35050-061, Gibco™), 1X antibiotic-antimycotic (Cat. No. 15240-062, 100 unit/mL penicillin, 100 μg/mL streptomycin, and 250 ng/mL amphotericin B, Gibco™) and 10% Fetal Bovine Serum (Gibco™) at 37 °C in a 5% CO_2_ humidified atmosphere. After reaching confluence, the cells were trypsinized using trypsin/EDTA (Cat. No. 25200-072, Gibco™) at a 1:3 ratio. Cells from passage 3–6 were used in the experiments. Four donor cell lines were used in the experiments.

In the cell differentiation assays, the cells were maintained in adipogenic medium [23], which was growth medium supplemented with 0.1 mg/mL insulin (Cat. No. I1882, Sigma-Aldrich), 1 μM dexamethasone, 1 mM 3-isobutyl-methylxanthine (IBMX, Cat. No. I5879, Sigma-Aldrich), and 0.2 mM indomethacin (Cat. No. I7378, Sigma-Aldrich). For neurogenic differentiation, neurosphere culture was performed by seeding cells in a Petri-dish (Cat. No. 430166, Corning, NY, USA) and the cells were maintained in neurobasal medium (Cat. No. 21103-049, Gibco™) supplemented with 2% B-27™ (Cat. No. 17504044, Gibco™), 2mM L-glutamine, 1X antibiotic-antimycotic, 20 ng/mL bFGF (Cat. No. 13256-029, Invitrogen, MD, USA), and 20 ng/mL EGF [13].

### 2.2 Flow cytometry analysis

The expression of hematopoietic and mesenchymal stem cell surface markers was determined using flow cytometry. Briefly, single cell suspensions were obtained by trypsinization with trypsin/EDTA solution. Subsequently, the cells were stained with the following fluorescence conjugated antibodies: FITC conjugated anti-human CD44 (Cat. No. 555478, BD Bioscience Pharmingen, NJ, USA), PE-conjugated anti-human CD105 (Cat. No. 21271054, Immuno Tools, Friesoythe, Germany), FITC-conjugated anti-human CD90 (Abcam, USA), and PerCP-conjugated anti-CD45 (Cat. No. 21810455, Immuno Tools). Mean fluorescence intensity was examined using a FACS^Calibur^ Flow cytometer (BD Bioscience).

For cell cycle analysis, cells (50,000 cells) were seeded in 6-well-plates and maintained with or without bFGF (Cat. No. 13256-029, Invitrogen, 20 and 40 ng/mL) for 72 h. The cells were then harvested and fixed with 70% v/v ice-cold methanol (Cat. No. RP230-4, Honeywell, Ulsan, Korea). PI/RNase staining (Sigma-Aldrich) was performed for 30 min. The samples were further analysed with a FACS^Calibur^ Flow cytometer.

### 2.3 Whole transcriptome analysis

Whole transcriptome analysis was performed using the NextSeq 5000 desktop sequencing system at the Omics Sciences and Bioinformatics Center, Faculty of Science, Chulalongkorn University. The cells were treated with bFGF (10 ng/mL, Invitrogen, USA) for 24 h in normal growth medium. Subsequently, total RNA was isolated using a RNeasy kit (Cat. No. 74104, Qiagen, MD, USA). DNase treatment was performed in columns. Total RNA quantity and quality were examined using a Nanodrop and Aligent 2100 Bioanalyzer system. Subsequently, total RNA was processed using a Ribo-zero rRNA removal kit (Illumina, USA) and subjected to cDNA synthesis using a TrueSeq Stranded mRNA library prep kit (Illumina, USA). Library quality assurance was conducted using an Aligent 2100 Bioanalyzer system and Qubit 3.0 fluorometer. Sequencing was performed in a NextSeq 500 sequencing system. Read quality was checked, trimmed, and filtered with a FastQC and FastX-toolkit [24]. Reads were mapped with *Homo sapiens UCSC hg38* using TopHat2 [25,26]. FPKM estimation of reference genes and transcripts was performed using Cufflink2 [26]. Assembly of the novel transcriptome was analysed using Cufflink2 and Cuffmerge [26]. Variant calling was examined using an Isaac Variant caller [27]. Differentially expressed genes and isoforms were determined using Cuffdiff2 [28]. The differentially expressed genes were further analysed for pathway enrichment using network-based visual analytics for gene expression profiling, meta-analysis, and interpretation, NetworkAnalyst [29]. RNA sequencing raw data was uploaded and made publicly available in the NCBI Sequence Read Archive (SRP199447) and NCBI Gene Expression Omnibus (GSE131758).

### 2.4 Colony formation assay

Five hundred cells were seeded on 60-mm tissue culture dishes (Cat. No. 174888, Nunc™, ThermoFisher Scientific, MA, USA). The cells were maintained in growth medium with or without bFGF for 14 d. The culture medium was changed every 48 h. The cells were washed with phosphate buffered saline and fixed with 10% buffered formalin (Cat. No. 1039991000, Merck Millipore, Darmstadt, Germany) for 10 min. Colonies were stained with Coomassie blue (Sigma-Aldrich). In some experiments, the cells were pretreated with an FGFR (SU5402 20 μM, cat. No. 215542-92-3, Sigma-Aldrich) or PI3K inhibitor (LY294002 1.4 μM, cat. No. 440202, Calbiochem) 30 min prior to bFGF exposure and maintained in culture medium supplemented with the inhibitor and bFGF.

### 2.5 Real-time polymerase chain reaction

Total RNA was extracted using Trizol reagent (RiboEX™, Cat. No. 301-001, GeneALL®, Seoul, Korea). Quality and quantity of the isolated RNA were determined using a Nanodrop ONE (ThermoFisher Scientific). One microgram of total RNA was converted to complimentary DNA using the Improm-II™ Reverse Transcription system (Cat. No. A3800, Promega, WI, USA). Real-time polymerase chain reaction (PCR) was performed using a FastStart Essential DNA green Master kit (Cat. No. 6402712001, Roche Diagnostic, IN, USA) on a LightCycler96 real-time PCR system (Roche Applied Science, USA). *18S* expression levels were used as the reference. The expression values were normalized using the 2^ˆ−ΔΔCt^ method. The primer sequences used are listed in Supplementary Table 1.

### 2.6 Immunofluorescence staining

Cells were washed with phosphate buffered saline and fixed in 10% buffered formalin for 30 min. Subsequently, the samples were incubated in 10% v/v horse serum for 1 h. Primary antibody incubation was performed at 4 °C overnight using a Ki-67 monoclonal antibody (SolA15), eBioscience™, (Cat. No. 14-5698-82, ThermoFisher Scientific). Biotinylated rabbit anti-rat antibodies (Cat. No. Ab6733, Abcam, MA, USA) were employed as secondary antibody and the samples were stained with Strep-FITC (Cat. No. S3762, Sigma-Aldrich). Cell nuclei were counterstained with DAPI (Cat. No. 32670, Sigma-Aldrich). The staining was evaluated under a fluorescence microscope ZEISS Apotome.2 (Carl Zeiss, Jena, Germany). The negative control was performed by omitting the primary antibody incubation step.

### 2.7 Statistical analysis

The data were plotted as mean ± standard error. The statistical analysis was performed using Prism 8 (GraphPad Software, CA, USA). The Mann Whitney U test was utilized for two group comparison. For three or more group comparison, the Kruskal Wallis test was employed followed by pairwise comparison. A statistically significant difference was defined as *p* < 0.05.

### 2.8 Ethics approval and consent to participate

The study was approved by Human Research Ethics Committee, Faculty of Dentistry, Chulalongkorn University (Approval number 079/2018). The procedure was performed according to the Declaration of Helsinki. Informed consent was obtained.

## 3 Results

### 3.1 Isolated cell characteristics

**Figure 1:**
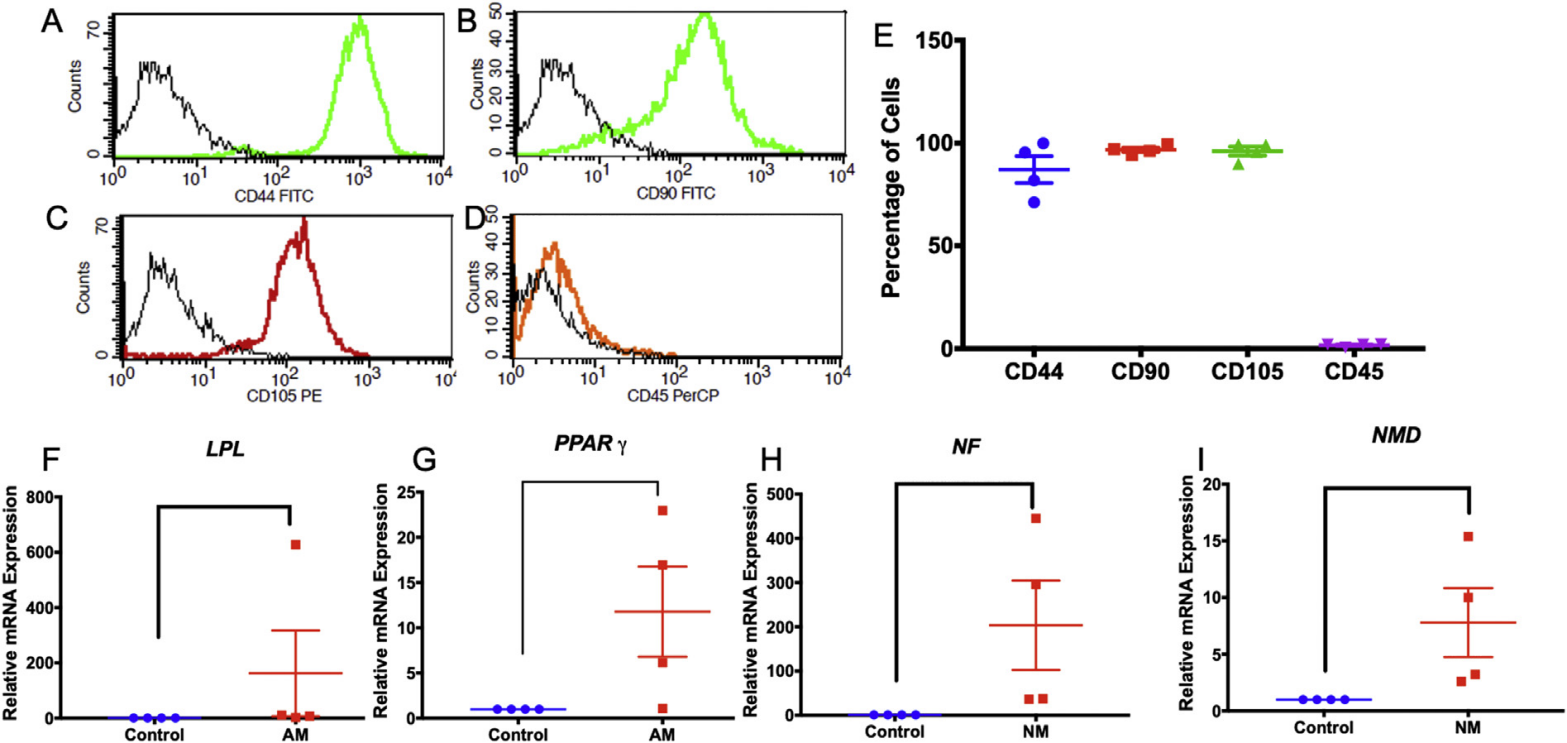
Characterization of the isolated cells. Stem cell surface markers were evaluated using flow cytometry (A–D). The percentage of cells expressing each surface marker (E). Cells were cultured in differentiation medium. The expression of differentiation markers was examined using real-time polymerase chain reaction (F–I) at day 8 of adipogenic induction and day 7 of neurogenic induction. Bars indicate a significant difference. (AM; adipogenic medium, NM; neurogenic medium).

The isolated cells (passage 3) expressed the mesenchymal stem cell surface markers CD44, CD90, and CD105, but not the hematopoietic stem cell marker CD45 (Figure 1A-E). At day 8 of adipogenic induction, the cells significantly increased *LPL* and *PPARγ* expression compared with the control (Figures 1F and 1G). Further, neurosphere culture induced neurogenic related gene expression (*NF* and *NMD*) compared with the undifferentiated control at day 7 (Figures 1H and 1I). These findings confirmed the mesenchymal stem cell characteristics of the isolated cells.

### 3.2 Transcriptome analysis of bFGF-treated SHEDs

Tables 1, 2, and 3 present the significantly differentially expressed genes, primary transcripts, and coding sequences that exhibited more than 1.5 Log 2 fold change. The entire list of the significantly differentially expressed genes, primary transcripts, and coding sequence are listed in Supplementary Table 2, 3, and 4, respectively. There was no difference in differential isoforms, differential splicing, differential coding output, or differential promotor use in bFGF-treated SHEDs compared with the control (data not shown).

**Table 1:** Gene-level differential expression of bFGF-treated SHEDs (more than 1.5 Log2 fold change).

Gene symbol	Gene name	Log2 fold change	*q* value
*SULT1B1*	Sulfotransferase family, cytosolic, 1B, member 1	2.76	0.030
*AKAP6*	A kinase (PRKA) anchor protein 6	2.42	0.045
*ALDH1A2*	Aldehyde dehydrogenase 1 family, member A2	2.15	0.012
*TMEM100*	Transmembrane protein 100	1.67	0.021
*SDPR*	Serum deprivation response	1.67	0.021
*TM4SF20*	Transmembrane 4 L six family member 20	-4.26	0.012
*INHBE*	Inhibin, beta E	-2.95	0.012
*CHAC1*	ChaC, cation transport regulator homolog 1 (E. Coli)	-2.92	0.012
*MRVI1*	Murine retrovirus integration site 1 homolog	-2.84	0.012
*WFDC1*	WAP four-disulfide core domain 1	-2.82	0.012
*TRIB3*	Tribbles homolog 3 (Drosophila)	-2.71	0.012
*ACTA2*	Actin, alpha 2, smooth muscle, aorta	-2.64	0.012
*SOST*	Sclerostin	-2.51	0.012
*COL15A1*	Collagen, type XV, alpha 1	-2.50	0.012
*ELN*	Elastin	-2.48	0.012
*KCND3*	Potassium voltage-gated channel, Shal-related subfamily member 3	-2.48	0.045
*COL11A1*	Collagen, type XI, alpha 1	-2.47	0.012
*ADM2*	Adrenomedullin 2	-2.46	0.012
*CLDN1*	Claudin 1	-2.38	0.021
*FLG*	Filaggrin	-2.33	0.021
*RAMP1*	Receptor (G protein-coupled) activity modifying protein 1	-2.28	0.012
*ACTG2*	Actin, gamma 2, smooth muscle, enteric	-2.14	0.012
*ITGA11*	Integrin, alpha 1	-2.01	0.012
*LBH*	Limb bud and heart development homolog (mouse)	-2.01	0.012
*LMOD1*	Leiomodin 1 (smooth muscle)	-1.75	0.012
*CNN1*	Calponin 1, basic, smooth muscle	-1.73	0.012
*PSAT1*	Phosphoserine aminotransferase	-1.69	0.012
*STC2*	Stanniocalcin 2	-1.69	0.049
*CTGF*	Connective tissue growth factor	-1.68	0.012
*KRTAP1-5*	Keratin associated protein 1-5	-1.67	0.012
*ASNS*	Asparagine synthetase (glutamine-hydrolyzing)	-1.63	0.012
*PCK2*	Phosphoenolpyruvate carboxykinase 2 (mitochondrial)	-1.58	0.045
*SLC7A11*	Solute carrier family 7 (anionic amino acid transporter light chain, xc-system) member 11	-1.58	0.045
*GPT2*	Glutamic pyruvate transaminase (alanine aminotransferase) 2	-1.57	0.012
*AMIGO2*	Adhesion molecule with Ig-like domain 2	1.52	0.012

**Table 2:** Primary transcript differential expression of bFGF-treated SHEDs (more than 1.5 Log2 fold change).

Gene symbol	Gene name	Log2 fold change	*q* value
*NPTX1*	Neuronal pentraxin I	1.50	0.037
*CHAC1*	ChaC, cation transport regulator homolog 1 (E. Coli)	-2.99	0.037
*ACTA2*	Actin, alpha 2, smooth muscle, aorta	-2.74	0.037
*TRIB3*	Tribbles homolog 3 (Drosophila)	-2.73	0.037
*COL15A1*	Collagen, type XV, alpha 1	-2.65	0.037
*ELN*	Elastin	-2.53	0.037
*SOST*	Sclerostin	-2.51	0.037
*LINC01125*	Chromosome 2 open reading frame 92	-2.48	0.037
*ADM2*	Adrenomedullin 2	-2.46	0.037
*RAMP1*	Receptor (G protein-coupled) activity modifying protein 1	-2.25	0.037
*ACTG2*	Actin, gamma 2, smooth muscle, enteric	-2.14	0.037
*ITGA11*	Integrin, alpha 1	-2.10	0.037
*LBH*	Limb bud and heart development homolog (mouse)	-1.94	0.037
*CNN1*	Calponin 1, basic, smooth muscle	-1.78	0.037
*STC2*	Stanniocalcin 2	-1.75	0.037
*LMOD1*	Leiomodin 1 (smooth muscle)	-1.75	0.037
*PSAT1*	Phosphoserine aminotransferase	-1.69	0.037
*CTGF*	Connective tissue growth factor	-1.68	0.037
*NUPR1*	Nuclear protein, transcriptional regulator, 1	-1.67	0.037
*KRTAP1-5*	Keratin associated protein 1-5	-1.66	0.037
*ASNS*	Asparagine synthetase (glutamine-hydrolyzing)	-1.61	0.037

**Table 3:** Coding sequence differential expression of bFGF-treated SHEDs (more than 1.5 Log2 fold change).

Gene symbol	Gene name	Log2 fold change	*q* value
*CHAC1*	ChaC, cation transport regulator homolog 1 (E. Coli)	-2.95	0.035
*WFDC1*	WAP four-disulfide core domain 1	-2.84	0.035
*ACTA2*	Actin, alpha 2, smooth muscle, aorta	-2.75	0.035
*TRIB3*	Tribbles homolog 3 (Drosophila)	-2.65	0.035
*SOST*	Sclerostin	-2.51	0.035
*ADM2*	Adrenomedullin 2	-2.46	0.035
*RAMP1*	Receptor (G protein-coupled) activity modifying protein 1	-2.25	0.035
*ACTG2*	Actin, gamma 2, smooth muscle, enteric	-2.13	0.035
*ITGA11*	Integrin, alpha 1	-2.13	0.035
*LBH*	Limb bud and heart development homolog (mouse)	-1.96	0.035
*LMOD1*	Leiomodin 1 (smooth muscle)	-1.83	0.035
*CNN1*	Calponin 1, basic, smooth muscle	-1.78	0.035
*CTGF*	Connective tissue growth factor	-1.68	0.035
*NUPR1*	Nuclear protein, transcriptional regulator, 1	-1.67	0.035
*KRTAP1-5*	Keratin associated protein 1-5	-1.67	0.035
*ASNS*	Asparagine synthetase (glutamine-hydrolyzing)	-1.65	0.035
*PSAT1*	Phosphoserine aminotransferase 1	-1.63	0.035
*AMIGO2*	Adhesion molecule with Ig-like domain 2	-1.51	0.035

**Figure 2:**
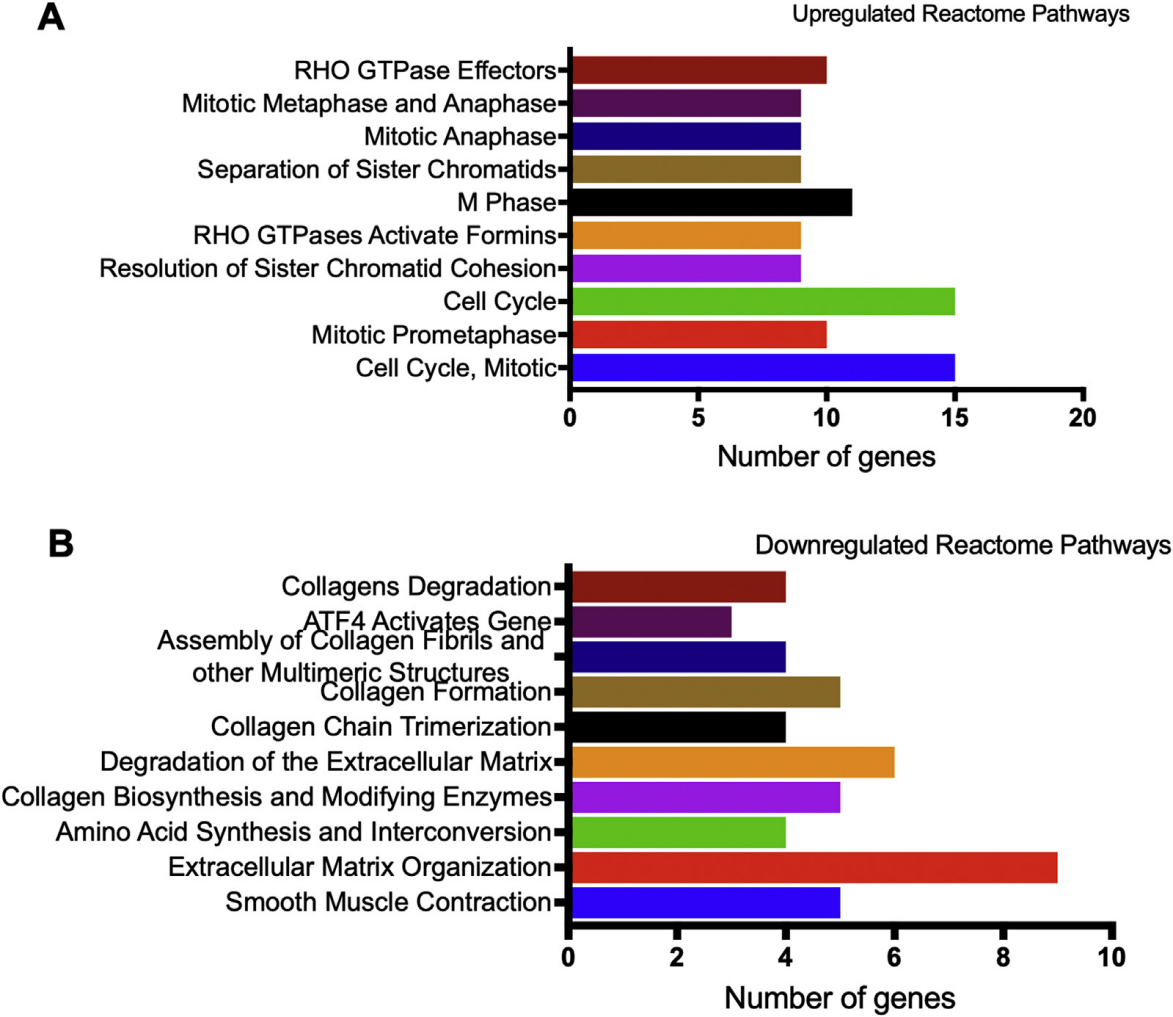
Pathway enrichment analysis. The reactome pathways for the upregulated genes (A) and downregulated genes (B) in bFGF-treated SHEDs.

**Figure 3:**
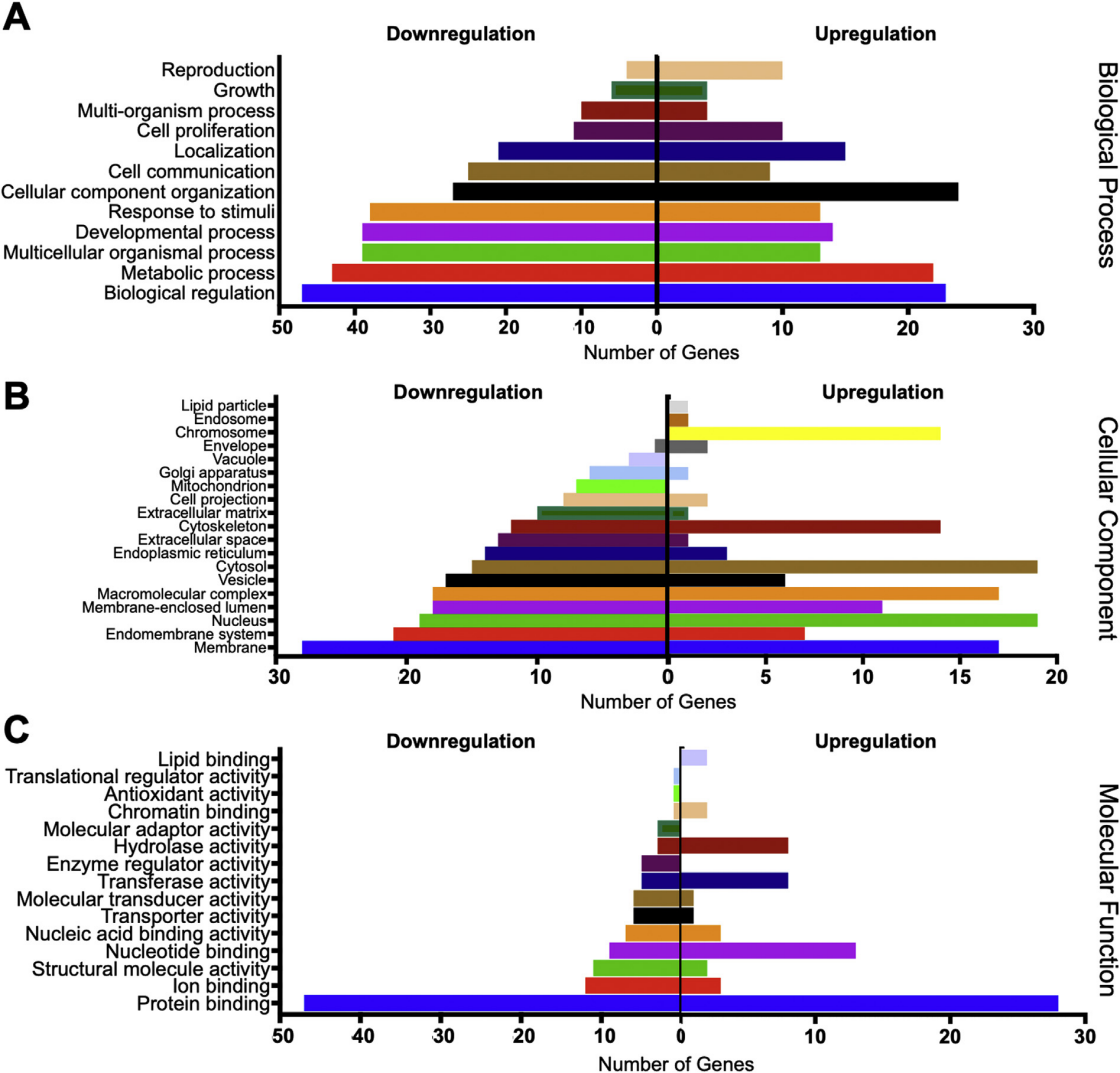
Gene ontology analysis. Gene ontology-based up- and downregulated genes of bFGF-treated SHEDs in biological process (A), cellular component (B), and molecular function (C) categories.

The bioinformatic analyses of the pathway enrichment of significantly differentially expressed genes are illustrated in Figures 2 and 3. The reactome pathways enrichment analysis demonstrated that the upregulated genes in bFGF-treated SHEDs were related to cell cycle related pathways, including cell cycle, mitotic, M Phase, and mitotic anaphase (Figure 2A). In contrast, the downregulated genes were categorized in the extracellular matrix related pathways, such as collagen degradation, collagen formation, extracellular matrix degradation, and extracellular matrix organization (Figure 2B). Gene ontology analysis showed that the majority of the upregulated genes were categorized in biological regulation and cellular component organization, while most of the downregulated genes were in biological regulation (Figure 3A). For cellular component evaluation, the upregulated genes were mostly in the nucleus and cytosol categories, however, the downregulated genes were in the membrane category (Figure 3B). Lastly, both up- and downregulated genes were in the protein binding category when analysed based on molecular function ontology (Figure 3C).

### 3.3 bFGF enhanced cell proliferation in SHEDs

**Figure 4:**
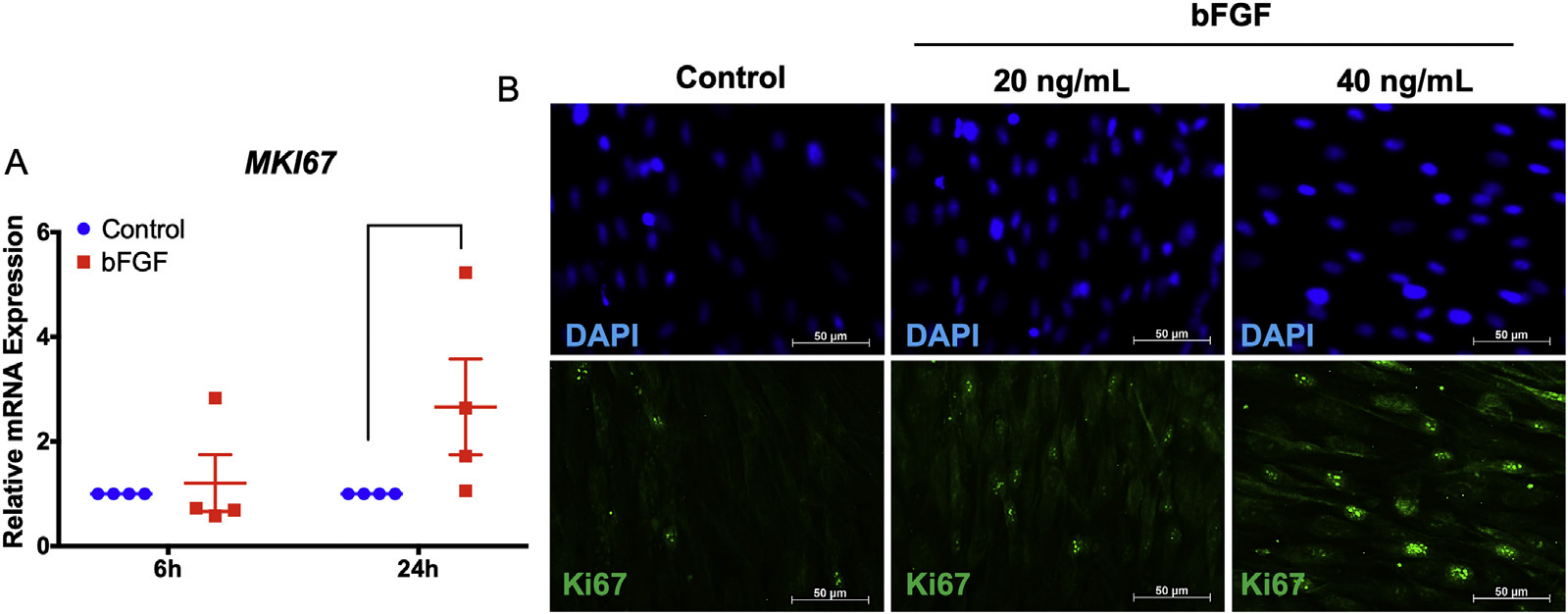
bFGF promoted Ki67 expression. SHEDs were treated with bFGF (20 ng/mL) and the mRNA expression of *MKI67* was examined using real-time polymerase chain reaction at 6 and 24 h after treatment (A). Ki67 protein expression was evaluated using immunofluorescence staining at 24 h after treatment (B). Bars indicate a significant difference.

**Figure 5:**
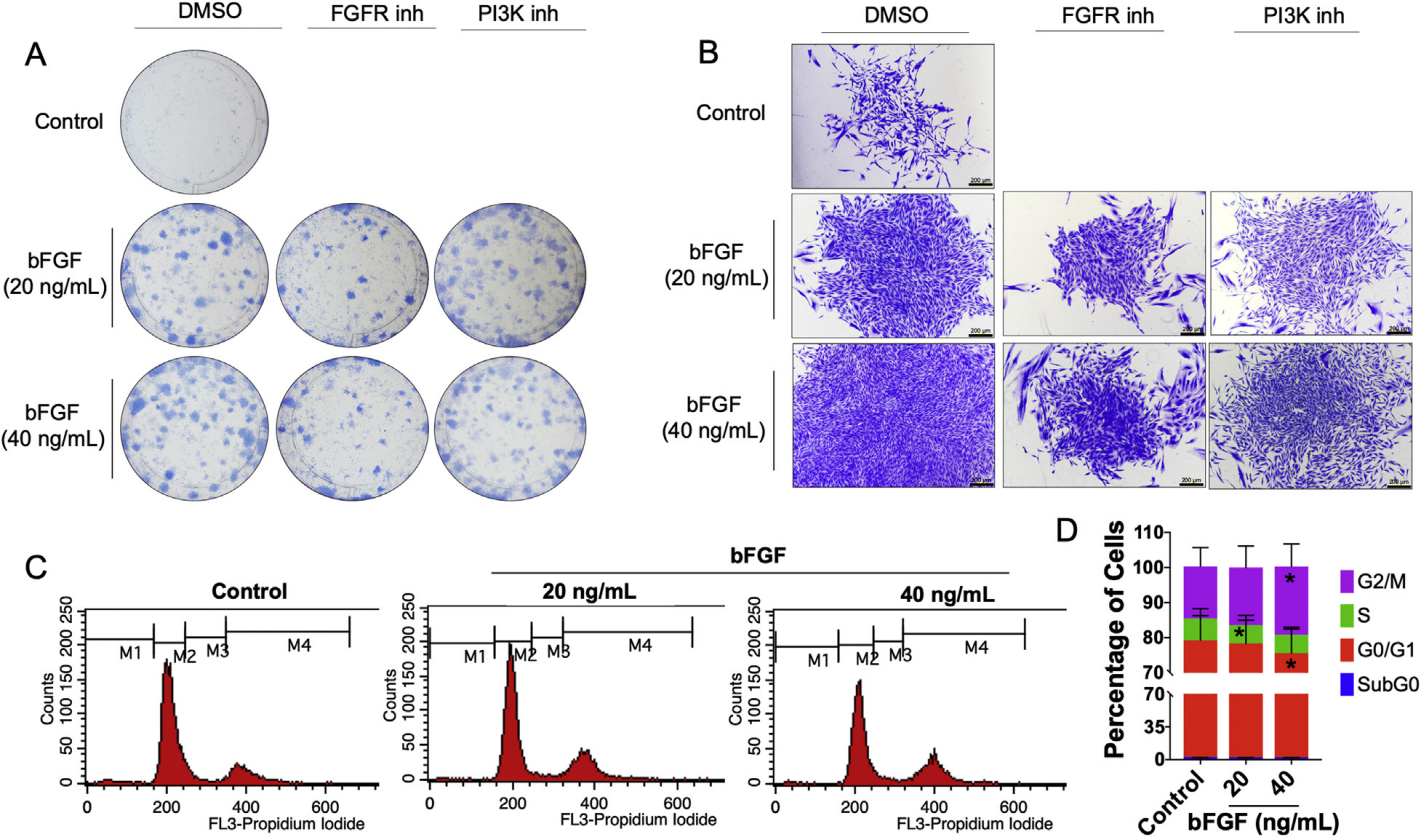
bFGF promoted SHEDs proliferation. Colony formation was stained with Coomassie blue at day 14 (A and B). In some conditions, cells were pretreated with an FGFR or PI3K inhibitor for 30 min prior to bFGF exposure. Further, the cells were maintained with or without the inhibitor and bFGF for 14 days. Black bars in (B) indicate 200 μm. Cells were treated with bFGF for 72 h and subsequently stained with PI/RNase. Cell cycle analysis was performed using flow cytometry. DNA histograms (C). The percentage of cell cycle subpopulations (D). Asterisks indicate a significant difference compared with the same subpopulation group in the control.

The upregulation of the proliferation marker gene, *MKI67*, was validated using real-time PCR. Although bFGF did not influence *MKI6*7 mRNA expression at 6 h, a significant increase in *MKI6*7 mRNA levels was observed in the bFGF-treated cells at 24 h (Figure 4A). Correspondingly, bFGF upregulated Ki67 protein expression at 24 h as determined by immunofluorescence staining (Figure 4B). Furthermore, bFGF supplementation enhanced colony formation in SHEDs at day 14 (Figure 5A). Colony number, size, and cellular density were increased in a dose-dependent manner (Figures 5A and 5B). This inductive effect was attenuated by an FGFR or PI3K inhibitor. The FGFR inhibitor markedly decreased colony number and size. The PI3K inhibitor slightly decreased colony number and size, while a marked decrease in cellular density was observed compared with the control vehicle. Moreover, bFGF treatment significantly increased the G2/M and decreased the G0/G1 population in SHEDs (Figures 5C and 5D).

## 4 Discussion

Despite the known mitogenic effect of bFGF on various stem cells including SHEDs, this present study is the first report on the transcriptome profile of bFGF-regulated SHEDs using a high throughput RNA sequencing technology. We found corresponding bFGF-induced differentially expressed genes, primary transcripts, and coding sequences. RNA sequencing generates both qualitative and quantitative information [30]. Unlike microarray, a high throughput RNA sequencing technique is not based on RNA and immobilized probe hybridization [30]. Thus, the bias from probe selection is diminished and low expression targets can also be detected [31,32]. A previous report demonstrated that the RNA sequencing technique was more beneficial for transcriptome analysis compared with microarray because the low abundance transcripts, isoforms, and genetic variants can be identified [32]. In the present study, differential isoforms, splicing, coding output, and promotor analyses were also performed. However, no significant differences were found in these assays. However, these analyses provided additional information to better understand the biological regulation of SHEDs by bFGF.

Studies have shown that bFGF influences both stemness maintenance and differentiation of various stem cells. bFGF upregulated the expression of pluripotent stem cell markers in SHEDs [9,10] and bFGF-induced REX1 expression was regulated by FGFR and Akt pathway [9]. Roles of bFGF in stem cell differentiation are also reported. Our previous reports demonstrated that bFGF addition in osteogenic induction medium resulted in the decrease of mineral deposition in SHEDs [33]. Correspondingly, endogenous bFGF inhibition resulted in the increase of mineral deposition in SHEDs [1]. For neurogenic differentiation, bFGF addition in neurogenic induction medium led to the increased size of neurospheres and the upregulation of neurogenic markers in human dental pulp stem cells [13]. Interestingly, roles of bFGF on7-20 adipogenic differentiation is stage dependent. In this regard, bFGF supplementation in early induction time point (first 2 days) led to the enhancement of adipogenic differentiation while the supplementation in later time points resulted in the inhibitory effects [34]. Taken all these evidences together, bFGF may play crucial role in both proliferation and differentiation of stem cells depending on cell types and stages as well as culture conditions.

Our results of the gene-level transcriptome demonstrated that bFGF-treated SHEDs increased their expression of genes in the cell cycle regulation pathways, while decreasing the expression of genes related to the extracellular matrix pathways. Correspondingly, bFGF promoted SHEDs colony forming unit ability as shown by increased colony number, size, and cellular density. Results confirmed the known information regarding the mitogenic effects of bFGF on SHEDs as previously reported by our group [1,10]. Moreover, the present study also revealed that bFGF regulated cell cycle and Ki67 expression. Further, we also identified that mitogenic effects of bFGF occurred via the PI3K pathway. Besides, other bFGF regulated pathways were also identified by transcriptome analyses. Further validation experiments will be indeed required to prove the influence of bFGF on those pathway regulations in SHEDs.

Previous studies have demonstrated the influence of bFGF on proliferation in various cell types, including bone marrow derived mesenchymal stem cells, umbilical cord blood mesenchymal stem cells, and Wharton's jelly mesenchymal stem cells [35,36,37]. In addition, bFGF treatment promoted cell proliferation in bone marrow-derived mesenchymal stem cells from diabetic mice [38]. Our previous study demonstrated that bFGF-induced SHED proliferation depends on cell density [10]. bFGF promoted cell proliferation at low cell density [10]. In this regard, bFGF enhanced colony numbers and cell density in colonies compared with the control [10]. However, bFGF did not alter proliferation when SHEDs were cultured at high densities [10]. It has been shown that FGFR expression was cell density-dependant, which influenced the cells' response to bFGF.

Previous report demonstrated that bFGF (range from 10-40 ng/mL) promoted angiogenesis in dose-dependent manner *in vitro* [39]. In human embryonic stem cells, bFGF at 10 ng/mL exhibited higher effects in the pluripotent marker expression than the higher doses while the effect of cell proliferation was not significant difference among tested doses in early cell passages [40]. On the contrary, bFGF (10–40 ng/mL) promoted proliferation in murine pre-osteoblast cell line in dose dependent manner [41]. bFGF at 40 ng/mL exhibited the highest inductive effect on alkaline phosphatase enzymatic activity in murine pre-osteoblast cell line [41]. However, it has been reported that low concentration (5–20 ng/mL) of bFGF promoted cell proliferation in bone marrow-derived MSCs and while high concentration (40 ng/mL) inhibited [42]. Thus, the optimal concentration of bFGF on specific cell types is indeed required for investigation. The present study utilized 2 different concentrations of bFGF (20 and 40 ng/mL) to observe the effect of dose and results demonstrated that bFGF enhanced Ki67 expression, colony forming unit, and cell cycle control in dose dependent manner.

When bFGF binds to its receptor, receptor phosphorylation occurs and subsequently triggers various intracellular signalling cascades, including the JAK/STAT, PLCγ, PI3K, and MEK pathways [8]. The present study found that bFGF promoted SHED colony forming unit number via the FGFR and PI3K pathways. It has been previously shown that bFGF induced neuronal differentiation in human dental pulp stem cells through the FGFR and PLCγ signalling pathways [13]. In SHEDs, bFGF inhibited mineral deposition *in vitro* via the ERK pathway [18]. Corresponding, our previous report showed that the bFGF-attenuated alkaline phosphatase enzymatic activity and mineral deposition were rescued by pretreating the SHEDs with a MEK inhibitor [43]. In contrast, the addition of a P38 inhibitor to bFGF-treated SHEDs in osteogenic medium resulted in markedly decreased mineralization [18]. Further, bFGF controlled pluripotent marker gene expression, *REX1,* in SHEDs via the FGFR/Akt pathways [9]. Taken together, bFGF may utilize different intracellular signalling pathways to control specific biological processes in SHEDs.

Ki67 is a proliferative marker and is expressed during the cell cycle [44]. In our study, a significant increase in *MKI6*7 mRNA and Ki67 protein expression was observed in the bFGF-treated cells. In addition, the G2/M subpopulation was higher in the bFGF-treated cells compared with the control. It has been reported that bFGF treatment in cardiac fibroblasts rescue the TGF-β1-suppressed cell proliferation by increasing Ki67 expression [37]. Moreover, the G2/M subpopulation was markedly increased, while the G0/G1 subpopulation was decreased in bFGF-treated human periodontal ligament stem/progenitor cells [45]. In contrast, bFGF inhibited cell proliferation and cell cycle progression in several cancer cell types, including adrenocortical carcinoma, breast cancer, and neuroepithelioma [46,47,48]. These results confirm the regulation of SHED cell proliferation by bFGF.

At the gene expression level, bFGF downregulated extracellular matrix pathway genes. *COL5A1, COL8A2, COL11A1,* and *COL15A1* mRNA levels were decreased in bFGF-treated SHEDs. Our previous report also demonstrated that bFGF significantly inhibited *COL1A1* mRNA expression in SHEDs at day 1, 3, and 7 after osteogenic induction [19]. Similarly, bFGF attenuated TGF-β1-induced *COL1A1* and *COL3A1* expression in cardiac fibroblasts [37]. In human periodontal ligament stem/progenitor cells, bFGF treatment dramatically downregulated *COL1A1, COL3A1, ACTA2,* and *FBN1* mRNA levels [45]. The influence of bFGF on collagen genes led to a hypothesis that bFGF acts as an anti-fibrotic agent in several cell types [49]. Furthermore, bFGF also inhibited members of the two other core categories of ECM molecules, glycoproteins (FBN2 and ELN) and proteoglycans. However, the effect of bFGF on SHEDs in regulating extracellular matrix genes remains unknown. Further study will be required to elucidate this function.

Previous reports from our laboratory demonstrated that bFGF attenuated odonto/osteogenic differentiation in hDPSCs and SHEDs [1,13,19]. bFGF inhibited alkaline phosphatase enzymatic activity and mineral deposition in SHEDs [19]. The present transcriptome analysis data suggest several explanations for these findings. First, *ITGA11* was downregulated in bFGF-treated SHEDs. Similarly, *ITGA11* expression was decreased by bFGF in human dermal fibroblasts [49]. It was hypothesized that bFGF acts as an anti-fibrotic agent in human dermal fibroblast cells [49]. The expression and function of ITGA11 in dental pulp cells of both deciduous and permanent teeth have never been reported. However, ITGA11 mRNA and protein were detected in human periodontal ligament cells (hPDLs) and human gingival fibroblasts (hGFs) [50]. When seeding hPDLs and hGFs on collagen 1-coated surfaces, ITGA11 expression was localized at the edge of the cells in a focal contact-like pattern and was shown to participate in extracellular matrix contraction in both cell types [50]. In addition, shRNA knockdown of *ITGA11* in human hepatic cells reduced myofibroblast differentiation [51]. The impaired myofibroblast phenotypes, including cell adhesion, cell migration, and collagen I contraction were observed in the *ITGA11* knockdown cells [51]. *Itga11* knockout in MC3T3-E1 cells resulted in reduced osteogenic differentiation, as determined by a significant decrease in *Dmp1* mRNA expression and mineral deposition *in vitro* [52]. Second, bFGF inhibited *CTGF* expression in SHEDs. CTGF has been shown to regulate reparative dentin formation. This molecule promoted cellular adhesion in murine pre-osteoblasts and *in vitro* mineralization in human dental pulp cells [53,54]. Moreover, adding CTGF to osteogenic medium stimulated osteogenic marker gene expression and mineralization in murine vascular smooth muscle cells [55]. ITGA11 and CTGF downregulation along with the downregulated extracellular matrix genes may explain the anti-fibrotic effects of bFGF. This effect should be explored more thoroughly in future studies, especially in the dental pulp.

Lastly, *ATF4* downregulation in bFGF-treated SHEDs may also participate in the mechanism by which bFGF attenuates osteogenic differentiation in SHEDs. It has been shown that reduced ATF4 expression corresponded with decreased osteogenic differentiation potency in human periodontal ligament stem cells [56]. In addition, miRNA targeting ATF4 negatively influences osteogenic differentiation *in vitro* and bone formation *in vivo* [56,57]. Hence, *ITGA11, CTGF,* and *ATF4* downregulation in bFGF-treated SHEDs may participate in the mechanism(s) by which bFGF negatively controlled odonto/osteogenic differentiation in these cells. Further investigation is needed to confirm this hypothesis.

## 5 Conclusion

This study reveals the effects of bFGF on the whole transcriptome of SHEDs. In addition, bFGF upregulates genes in cell cycle progression, while attenuating the expression of genes related to extracellular matrix formation and remodelling in these cells. The present study also highlights transcriptomic profile of bFGF-treated SHEDs which shown the regulation of SHED self-renewal and cell proliferation.

## Declarations

### Author contribution statement

N. Nowwarote, J. Manokawinchoke and K. Kanjana: Performed the experiments; Analyzed and interpreted the data; Wrote the paper.

W. Sukarawan: Analyzed and interpreted the data; Contributed reagents, materials, analysis tools or data; Wrote the paper.

B. Fournier: Analyzed and interpreted the data.

T. Osathanon: Conceived and designed the experiments; Analyzed and interpreted the data; Wrote the paper.

### Funding statement

This work was supported by the Chulalongkorn Academic Advancement into Its 2nd Century Project and Faculty of Dentistry Research Fund, 10.13039/501100002873Chulalongkorn University (DRF 60016). T. Osathanon was supported by the 10.13039/501100004396Thailand Research Fund (TRF-RSA6180019). N. Nowwarote was supported by the Ratchadapisek Sompote Fund for Postdoctoral Fellowship, 10.13039/501100002873Chulalongkorn University.

### Competing interest statement

The authors declare no conflict of interest.

### Additional information

Data associated with this study has been deposited at NCBI Sequence Read Archive under the accession number SRP199447, and NCBI Gene Expression Omnibus under the accession number GSE131758.

Supplementary content related to this article has been published online at https://doi.org/10.1016/j.heliyon.2020.e04246.

## Appendix ASupplementary data

Suppl Table 1 Suppl Table 1

Suppl Table 2 Suppl Table 2

Suppl Table 3 Suppl Table 3

Suppl Table 4 Suppl Table 4
