# Supplementary material for: Transcriptome analysis of basic fibroblast growth factor treated stem cells isolated from human exfoliated deciduous teeth
Source: Heliyon. 2020 Jun 25;6(6):e04246. doi: 10.1016/j.heliyon.2020.e04246 (PMC7322690; doi:10.1016/j.heliyon.2020.e04246)
Supplement: Suppl Table 1 [file mmc1.docx]

**Supplementary Table 1.** Primer sequences

| **Gene** | **Forward** | **Reverse** | **Accession number** |
| --- | --- | --- | --- |
| ***18S*** | 5’-GGCGTCCCCCAACTTCTTA-3’ | 5’-GGGCATCACAGACCTGTTATT-3’ | NR003286.2 |
| ***MKI67*** | 5′ AGAAGAAGTGGTGCTTCGGAA 3' | 5′ AGTTTGCGTGGCCTGTACTAA 3′ | NM_001145966.1 |
| ***RUNX2*** | 5’ ATGATGACACTGCCACCTCTG 3’ | 5’ GGCTGGATAGTGCATTCGTG 3’ | NM001024630.3 |
| ***DSPP*** | 5’ ATATTGAGGGCTGGAATGGGGA 3’ | 5’ TTTGTGGCTCCAGCATTGTCA 3’ | NM014208.3 |
| ***LPL*** | 5’ GAGATTTCTCTGTATGGCACC 3' | 5' CTGCAAATGAGACACTTTCTC 3' | NM000237.2 |
| ***PPARG*** | 5′CCAGTGGTTGCAGATTACAAGTATG 3′ | 5′TTGTAGAGCTGAGTCTTCTCAGAATAATAAG 3' | NM138712.3 |
| ***NF*** | 5’ ACCCGACTCAGTTTCACCAG 3’ | 5’ CTCATCCTTGGCTTCCTCAG 3’ | NM006158.4 |
| ***NMD*** | 5’ CACTGATAACTCGCCGTCCT 3’ | 5’ CTCTTCAGCTTGGCTGCTCT 3’ | NM002045.3 |
